# Periodic Addition of Glucose Suppressed Cyanobacterial Abundance in Additive Lake Water Samples during the Entire Bloom Season

**DOI:** 10.4236/jwarp.2024.162009

**Published:** 2024-02-23

**Authors:** David Linz, Ian Struewing, Nathan Sienkiewicz, Alan David Steinman, Charlyn Gwen Partridge, Kyle McIntosh, Joel Allen, Jingrang Lu, Stephen Vesper

**Affiliations:** 1 United States Environmental Protection Agency, Cincinnati, Ohio, USA; 2 Annis Water Resources Institute, Grand Valley State University, Muskegon, USA; 3 Oak Ridge Institute for Science and Education, Oak Ridge, Tennessee, USA

**Keywords:** Glucose, Cyanobacteria, 16S Amplicon Sequencing, Microbial Community

## Abstract

Previously, we showed that prophylactic addition of glucose to Harsha Lake water samples could inhibit cyanobacteria growth, at least for a short period of time. The current study tested cyanobacterial control with glucose for the entire Harsha Lake bloom season. Water samples (1000 ml) were collected weekly from Harsha Lake during the algal-bloom season starting June 9 and lasting until August 24, 2022. To each of two 7-liter polypropylene containers, 500 ml of Harsha Lake water was added, and the containers were placed in a controlled environment chamber. To one container labeled “Treated,” 0.15 g of glucose was added, and nothing was added to the container labeled “Control.” After that, three 25 ml samples from each container were collected and used for 16S rRNA gene sequencing each week. Then 1000 ml of Harsha Lake water was newly collected each week, with 500 ml added to each container, along with the addition of 0.15 g glucose to the “Treated” container. Sequencing data were used to examine differences in the composition of bacterial communities between Treated and Control containers. Treatment with glucose altered the microbial communities by 1) reducing taxonomic diversity, 2) largely eliminating cyanobacterial taxa, and 3) increasing the relative abundance of subsets of non-cyanobacterial taxa (such as Proteobacteria and Actinobacteriota). These effects were observed across time despite weekly inputs derived directly from Lake water. The addition of glucose to a container receiving weekly additions of Lake water suppressed the cyanobacterial populations during the entire summer bloom season. The glucose appears to stimulate the diversity of certain bacterial taxa at the expense of the cyanobacteria.

## 1. Introduction

Cyanobacterial blooms are increasing worldwide [1], threatening aquatic ecosystems and drinking- and recreational-water safety [2] [3]. The long-term solution to the cyanobacterial bloom problem is the reduction of nutrient additions, especially forms of nitrogen and phosphorous, to the aquatic ecosystem. In the near-term, various methods and treatments have been investigated to control or reduce cyanobacterial blooms. These treatments include the use of aquatic plants to absorb the excess nutrients [4]. Biologically derived molecules have also been tested to control blooms [5] [6]. Algicidal bacteria [7], bacterial predation [8], viral infection [9], and fungal derived products [10] [11] have also been evaluated. Physical measures have also been evaluated, including coagulation/flocculation treatments [12] and photodegradation of the algal toxins [13]. Therefore, treatment is still challenging despite the very few effective options, like toxic chemicals.

High concentrations of hydroxyurea were tested in the past to control *Anabaena* [14]. Chemicals more commonly used today to control blooms are hydrogen peroxide and copper salts [15] [16]. The chemicals can be dangerous to handle and toxic. Hydrogen peroxide is dangerous to handle in large quantities and is an indiscriminate oxidizer, killing many organisms besides the cyanobacteria [17]. Copper salts are lethal agents for many organisms and can bioaccumulate in the ecosystem [18]. Recently, we demonstrated that the addition of glucose to freshwater samples could limit cyanobacterial growth and toxin production [19]. However, the test was conducted for only two weeks due to the limits of culture conditions.

In this study, we tested how the weekly addition of glucose to freshwater samples during the entire bloom season altered the cyanobacterial community. We demonstrate that the maintenance of glucose during the bloom season nearly eliminated cyanobacterial growth, even after the addition of freshly collected Lake water each week.

## 2. Materials and Methods

### 2.1. Study Site

The William H. Harsha Lake (hereafter, Lake) is an engineered reservoir created in 1978 located in southeast Ohio, as previously described in detail [20]. Harsha Lake covers an area of 8.7 km^2^ and drains from a watershed of 890 km^2^, with 64% of land used for agriculture and 26% comprised of forest cover [21]. The development of algal blooms in Harsha Lake has been studied by our team since 2015 [20] [21] [22]. Water collected from Harsha Lake during the 2022 bloom season was used for this study. Based on our experience in Harsha Lake studies, cyanobacterial populations start with N_2_-fixing *heterocystous cyanobacteria* in late May when N is low, and then transitions to non-N_2_-fixing *Microcystis*- and *Planktothrix*-dominated populations when N concentration is lifted after N2 fixation [23]. Harsha Lake can be described as a moderately eutrophic lake.

### 2.2. Collection of Harsha Lake Samples

Harsha Lake water samples were collected weekly, starting June 9 until August 24, 2022, using plastic water jugs, which were pre-rinsed using 5% hydrochloric acid and deionized water, to sample water from the surface (~0.5 m depth) at 2 locations, as previously described [20]. Each week, the samples from each location were combined to make a single 1000 ml composite sample.

### 2.3. Study Design

Two 7-liter polypropylene containers (8SFSPP, CAMBRO, Huntington Beach, CA) were used in this study. To each container was added 500 ml of the freshly collected Harsha Lake water. In addition, 0.15 g of glucose [D- (+) Glucose SigmaUltra, Sigma-Aldrich, St. Louis, MO] was added to the “Treated” container, but nothing was added to the “Control” container. Each container was covered with a transparent xerography sheet (Skillcraft, Greensboro, NC). The containers were mixed daily on a stir plate for 5 min during the study. To mimic the changing microbial populations in the Lake during the summer bloom season, fresh water samples (1000 ml) were collected each week from the two sampling locations, combined and mixed. Then, each week 500 ml was added to each of the two containers, just as was done initially. To maintain the glucose level in the Treated container, 0.15 g of glucose was also added each week.

The containers were incubated in an environmental chamber (Percival/166LLVL) with the following growth conditions: the light intensity was 44.02 μmol photons/m^2^/s (measured using a LICOR LI-1500) with a 16/8-hour light/dark cycle at a constant temperature of 25°C and ambient air-exchange. These conditions were not intended to reflect the daily outside environment of the Lake but to provide optimal conditions for plankton growth.

### 2.4. Weekly Measurement of Physical, Chemical and Biological Parameters of the Lake Water

EXO2 multi-parameter water quality sonde multiprobes (Yellow Springs Instruments Inc., Yellow Springs, OH, USA) were used to measure temperature, pH, oxidation-reduction potential, specific conductance, chlorophyll fluorescence, phycocyanin fluorescence and turbidity at the water surface. Sensors were calibrated following manufacturer’s guidance. Calibration drift was corrected as described [24]. Departures from calibration are assessed by comparing the sensor’s response in a standard solution to the known value of the standard solution following guidance regarding deviation limits and post-deployment corrections [24].

### 2.5. DNA Extraction and High-Throughput Sequencing

Each week, three 25-ml samples were collected from each container for sequencing. Each sample was filtered through a Durapore polyvinylidene fluoride (PVDF) filter, with 0.45 μm pore size (MilliPore, Foster City, CA). Each filter was inserted into a 1.5-ml bead-beating tube (MP Biomedicals, LLC, Santa Ana, CA) and stored at −80°C until extracted.

Each bead-beating tube with filter was recovered from the −80°C freezer, and 600 μl of the cell lysis Buffer RLT (QIAGEN, Valencia, CA) was added to each tube to prepare for DNA extraction. Filters were mechanically disrupted, and cells lysed using a Mini-Beadbeater-16 (BioSpec Products, Inc., Bartlesville, OK) twice for 30 sec and then centrifuged at 10,000 *g* for 3 min. The supernatant was then transferred to a new sterile tube, and the DNA was purified using the All-Prep DNA/RNA mini QIAGEN Kit (QIAGEN, Fredrick, MD) following the manufacturer’s instructions. The extracted DNA was eluted in 200 μL RNase-free water (Sigma-Aldrich, St. Louis, MO) for sequencing.

For amplicon sequencing, the v3-v4 hypervariable regions of the 16Sr RNA gene were targeted using primers described [25]. Library preparation and sequencing were performed, as described [26], with the following modifications. The first round of PCR was performed with 17 μL 1.1x Accuprime pfx supermix (Thermo-Fisher, Waltham, MA), 0.5 μL of each primer, and 2 μL of DNA. After gel confirmation of the amplification products, PCR products were cleaned with AMPure XP beads (Beckman Coulter, Brea, CA) and eluted in 10 mM Tris pH 8.5. PCR products were normalized to 20 ng/μL, and an index PCR was performed using Accuprime pfx supermix and Nextera indexes. The index PCR product was cleaned using AMPure XP beads and eluted in 10 mM Tris pH 8.5. Samples were normalized to a concentration of 4 nM and combined to make the final library. The library was sequenced using a 600 cycle V3 MiSeq sequencing kit (# MS-102–3003, Illumina, San Diego, CA, USA) according to the manufacturer’s protocol, using 2 × 300 paired-end sequencing.

### 2.6. 16S Cloning and Quantitative Real-Time PCR Amplification

A qPCR assay with forward CYAN108F and reverse primer CYAN377R were used to quantify the 16S rRNA gene of cyanobacteria [27]. The PCR was performed in a BioRad Thermocycler (MJ Research, Bio-Rad Laboratories, USA). The PCR mixture contained 2 μL of DNA template solution (~100 ng DNA), 2 μL of 10× PCR Buffer [100 mM Tris–HCl (pH 8.3), 500 mM KCl, 15 mM MgCl2], 0.5 μL of 2.5mM dNTP mixture, 0.25 μL (10 μM) of each primer, 2 μL of 1 mg/mL Bovine Serum Albumin (BSA) and 0.25 μL Taq DNA Polymerase (TaKaRa Biotechnology, Japan), and was adjusted to a final volume of 20 μL with Molecular Biology Grade sterile water (Corning, USA). The PCR was performed as follows: 94°C for 2 min, 30 cycles at 94°C for 1 min, 60°C for 1 min and 72°C for 1 min, and a final extension step at 72°C for 7 min. The PCR product was examined on 2% (w/v) agarose gels stained with GelStar^™^ Nucleic Acid Gel Stain (LONZA, Rockland, ME, USA). The cloned PCR amplicon was further incorporated into Invitrogen^™^ pCR^™^ 4-TOPO^™^ Vector using the manufacture’s TOPO TA cloning kit to be used as the plasmid standard.

Real-time PCR assays applied for the quantification of 16S rRNA gene of cyanobacteria with forward (CYAN108F) and reverse (CYAN377R) primers, as described by [28]. SYBR^®^ Green PCR was used on a QuantStudio^™^ 6 Flex System (Life Technologies Co.) to detect the abundance of the 16S rRNA gene in the Harsha Lake samples [19] [21] [22]. Each reaction (final volume: 20 μL) was composed of 10 μL of 2 × SYBR^®^ Green Master Mix (Life Technologies Co.), 0.25 μM of primers (Integrated DNA Technologies, Inc., Coralville, Iowa, USA), 2 μL of 1 mg/mL BSA, and 2 μL of template DNA. The following thermal cycling conditions were applied: 40 cycles of 95°C for 15 s, annealing temperatures of 56°C for 30 s, an extension step at 72°C for 30 s, and a hold step at 72°C for 5 min, followed by melt curve analysis. The DNA was quantified against the series of standards constructed in-house. The standard series of 16S were generated from *Microcystis* DNA isolated from the cloning process mentioned above. Each Harsha Lake sample was added on a qPCR plate, which included a six-point standard curve with target gene concentrations ranging from 10^6^ to 10^0^ copies·μL^−1^, using a tenfold serial dilution. PCR inhibition was manually checked by measuring 10-fold diluted DNA extracts using qPCR, and datapoints where significant PCR inhibition was detected were removed by following an established protocol [29].

### 2.7. Amplicon Processing

Raw demultiplexed reads, with adapters removed, were then processed using the software suite QIIME 2 2021.4.0 [30]. Raw sequence data were quality filtered and denoised with DADA2 (via q2-dada2) [31]. Chimeras were removed using DADA2. Taxonomy was assigned to amplicon sequence variants (ASVs) based on the Silva 138 SSU reference using the q2-feature-classifier [32] and classify-sklearn naive Bayes taxonomy classifier ¨ [33] [34]. Qiime2 artifacts were then moved to R v4.1.2 using the qiime2R package for further analysis [35]. All data have been deposited in the National Center for Biotechnology Information (NCBI) sequence read archive at accession number: PRJNA972685.

### 2.8. Sequence Data Analysis

Analysis of the final sequence dataset was performed in R v4.1.2 [36] using the packages phyloseq (v1.38.0) [37], vegan (v2.5.7) [38], and ggplot2 (v3.3.5) [39]. Samples were initially pruned of non-bacterial or unidentified taxa. Replicates for each sample were initially examined and, after determining consistency among replicates, merged for subsequent analysis. For certain components of our analysis, low abundance taxa were removed from the dataset. Bray-Curtis between-sample distances were computed. Distance matrices were then used to cluster samples using non-metric multidimensional scaling (NMDS). Analysis of Similarities (ANOSIMs) on the distance matrices were used to test for statistically significant differences in the bacterial composition and diversity between sample groups in control versus glucose treatment containers [40]. A student’s t-test was used to test for differences in observed taxa between Treated and Control containers. Where appropriate, assumptions of normality and homoscedasticity were validated visually (with Q-Q plots) and statistically (using Levene’s test for equality of variance) to determine appropriate tests.

## 3. Results

### 3.1. Changes in the Physical, Chemical and Biological Parameters of the Lake Water

The changes in the physical, chemical and biological parameters of the Lake at the time of sampling each week are shown in Table 1. In general, nutrient concentrations were higher early in the summer and decreased thereafter. This is reflected in higher chlorophyll and phycocyanin levels early in the summer, which generally decreased thereafter. A similar trend was also observed in the concentration of disolved oxygen and turbidity. The temperature and pH of the Lake water also declined over the summer.

### 3.2. Changes in Community Structure in Treated and Control Containers

**Figure 1.**
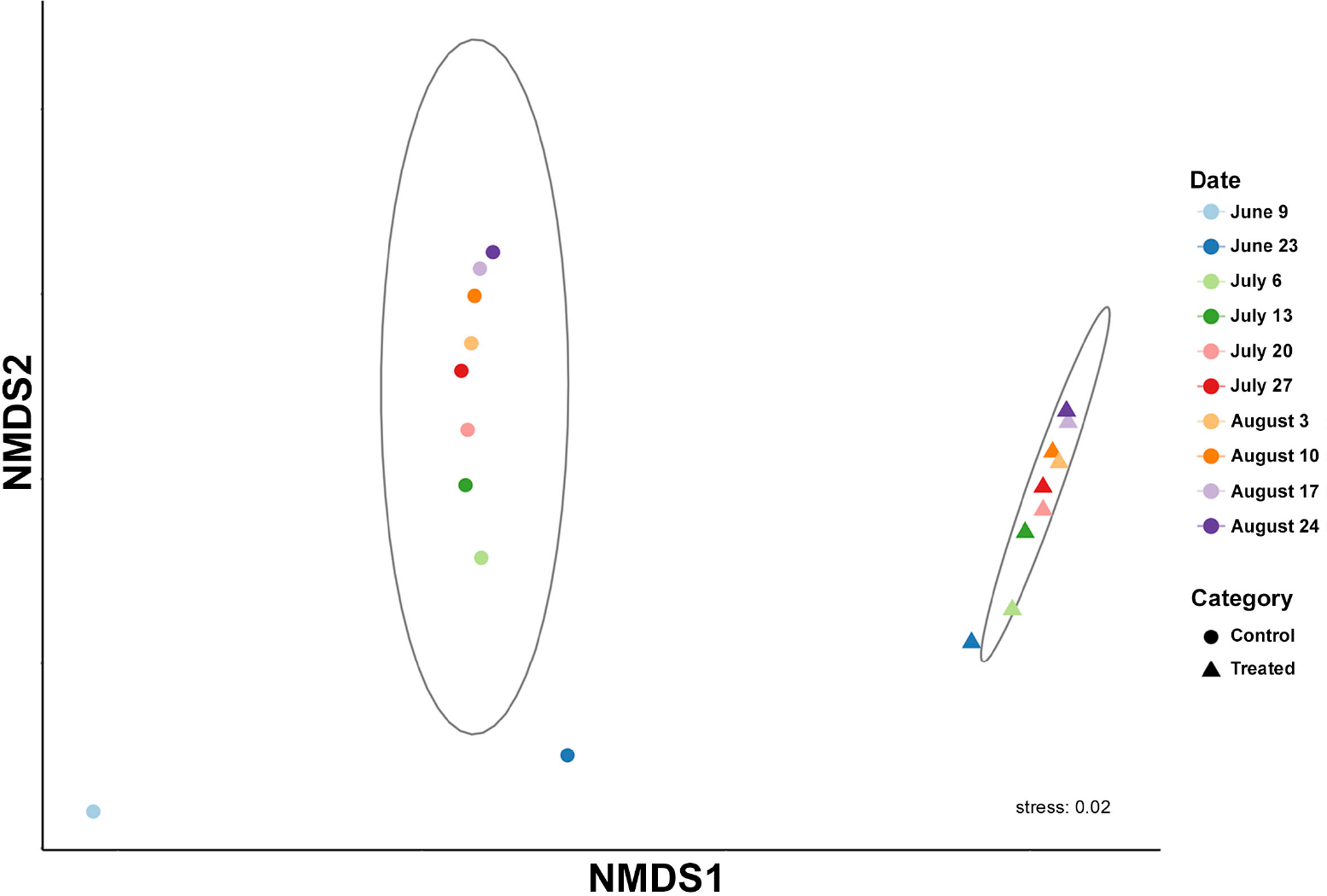
Beta diversity of microbial community composition in treated and control conditions across dates shown via non-metric multidimensional scaling (NMDS) plot. Category of treatment is indicated by shape and date is indicated by color.

**Figure 2.**
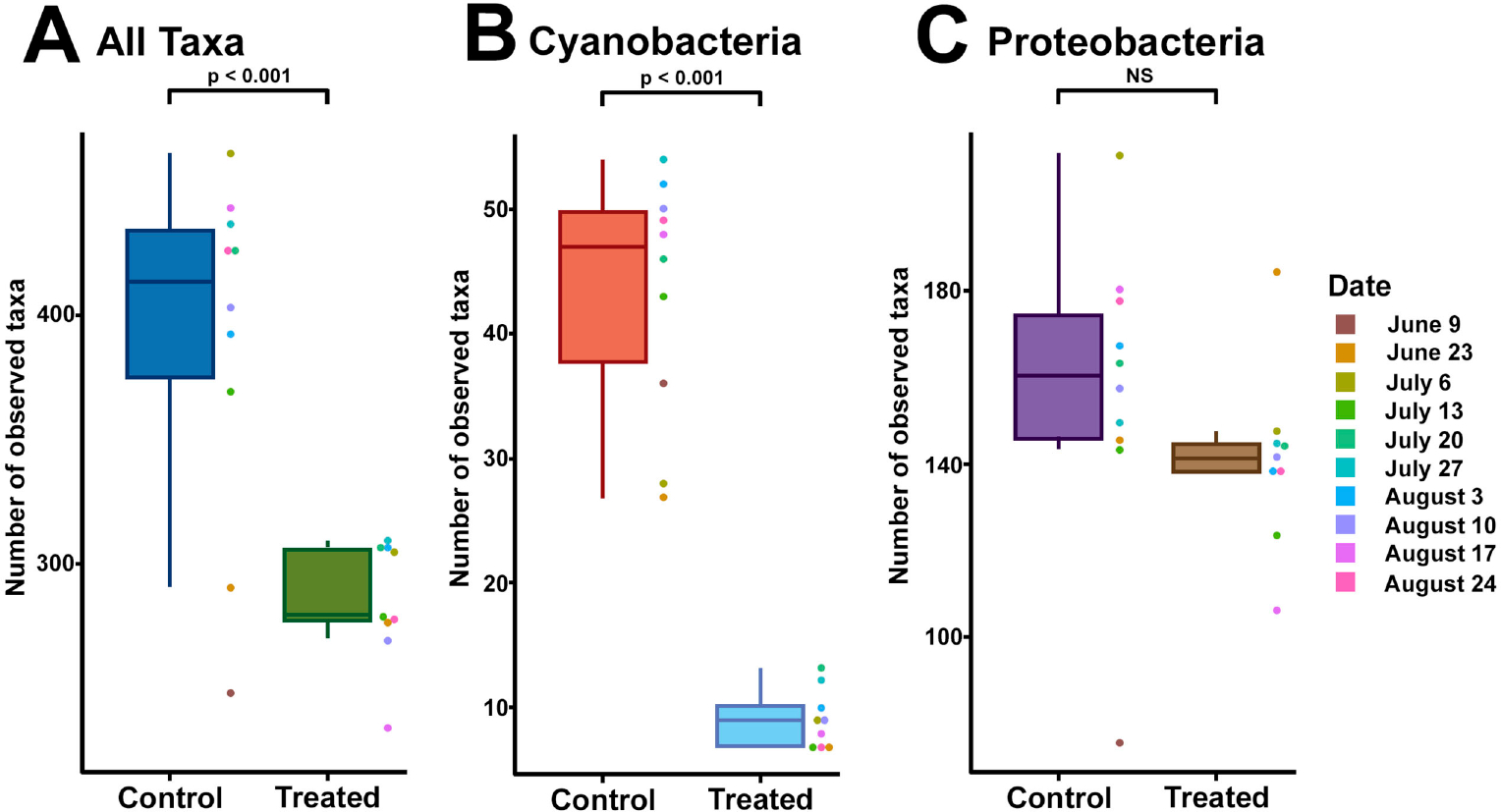
Raw observed taxa in control and treatment conditions with date indicated by color (legend on right applies to all panels). (A) All taxa. (B) Cyanobacteria. (C) Proteobacteria. Significance of Student’s T-test is shown above. NS = not significant.

To explore possible differences in general microbial community composition between Treated and Control containers and among dates (beta diversity), we performed ordination using non-metric multidimensional scaling (NMDS). Across sample dates, bacterial communities in the Treated and Control containers were significantly different in their composition (Bray-Curtis, ANOSIM; R = 0.956, p < 0.001) (Figure 1). The single control sample from June 9^th^ taken directly from Lake Harsha also deviated considerably from the remainder of our Control samples (Figure 1; light blue dot in lower left corner). We next examined distributions in raw observed taxa between our control samples and treated samples and found a significant reduction in overall taxa in our treated samples (Figure 2A).

**Figure 3.**
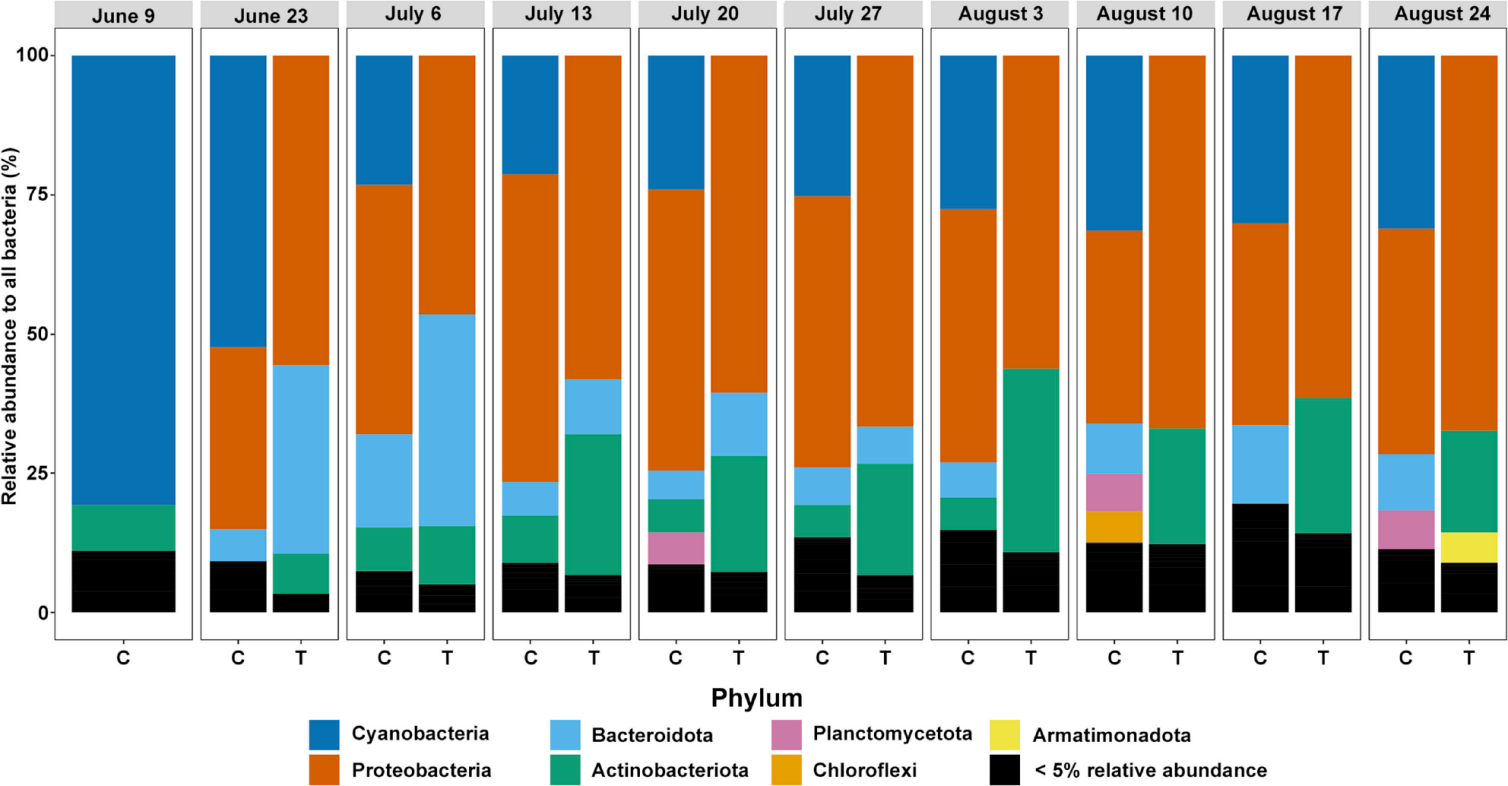
Relative abundance of bacterial phyla occurring above 5% (compared to all bacterial species) in each experimental condition (control = C, treatment = T) at each time point sampled. Samples are grouped by date (top).

To investigate the specific taxa contained within the two experimental conditions, we examined the relative abundance of taxa within both Control and Treated containers across sampling dates. First, we explored taxa at the phylum level (Figure 3). We found that, in line with our NMDS, the first control sample taken from Lake Harsha contained over 75% Cyanobacteria. Following this initial collection, the control sample’s community composition changed—con- taining larger portions of Proteobacteria while maintaining modest proportions of Cyanobacteria and other phyla. In contrast, treated samples contained <5% relative abundance of cyanobacteria while concomitantly increasing proportions of Proteobacteria, Bacteroidota, and Actinobacteriota.

**Figure 4.**
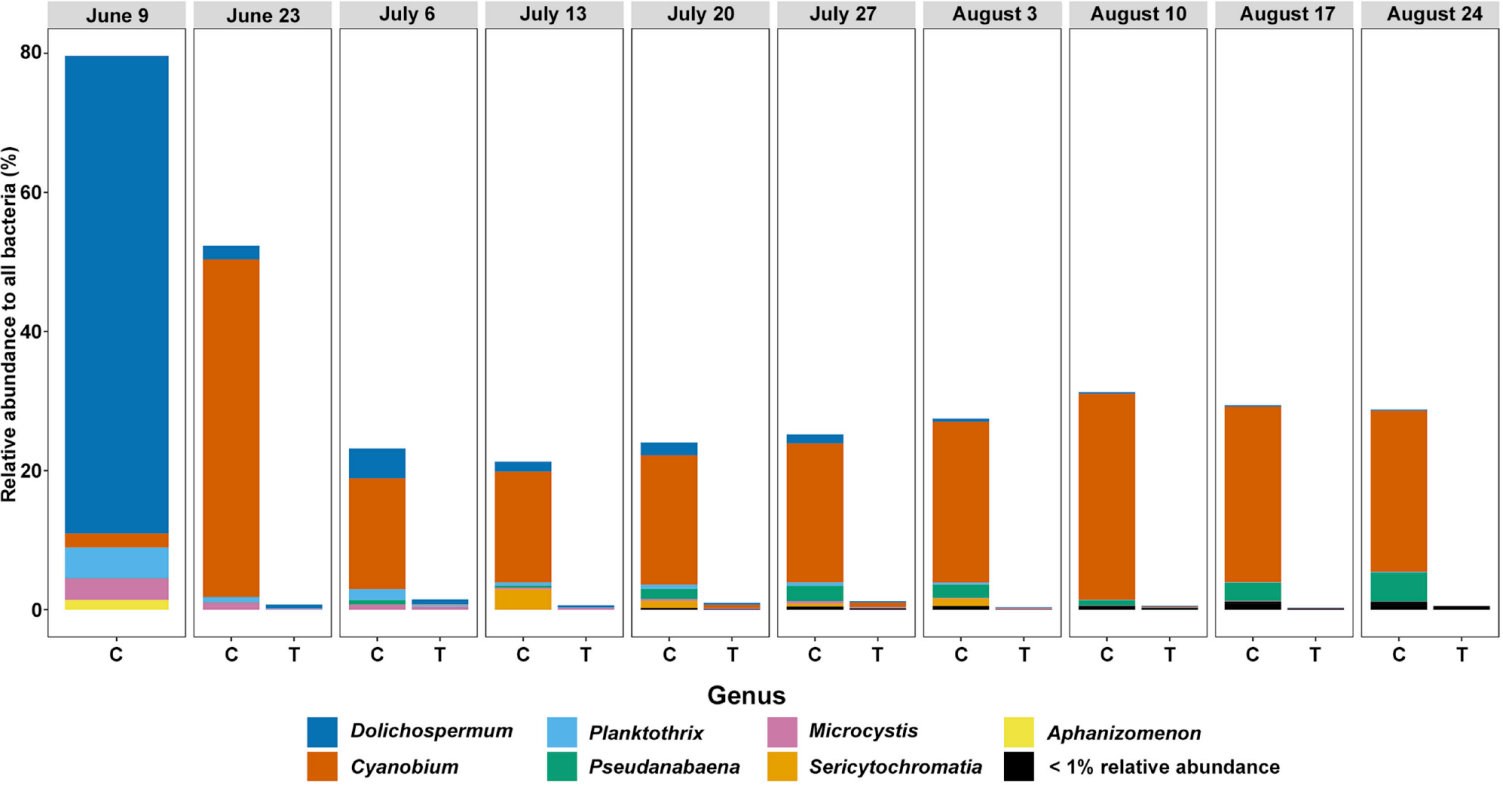
Relative abundance of cyanobacterial genera occurring above 1% (compared to all bacterial species) in each experimental condition (control = C, treatment = T) at each time point sampled. Samples are grouped by date (top).

**Figure 5.**
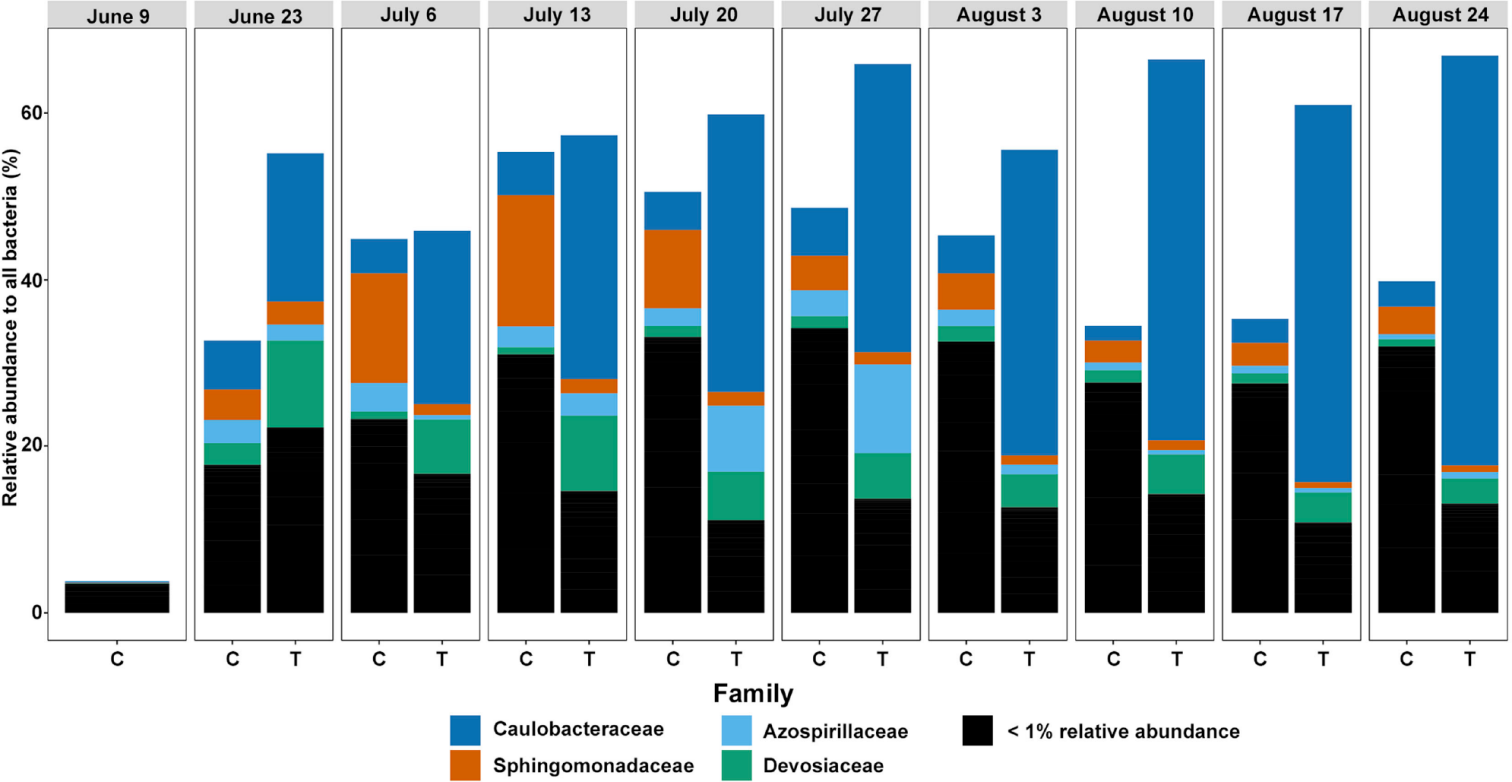
Relative abundance of proteobacterial families occurring above 1% (compared to all bacterial species) in each experimental condition (control = C, treatment = T) at each time point sampled. Samples are grouped by date (top).

We next examined the Cyanobacterial genera (Figure 4) and Proteobacterial families (Figure 5) across dates and treatments to understand compositional changes occurring within key phyla. Within the Cyanobacteria, our initial Lake Harsha sample (June 9^th^) was dominated by *Dolichospermum*, and after transitioning into our experimental setup, these genera shifted, becoming dominated by *Cyanobium* spp. Importantly, Cyanobacteria were almost completely absent from the Treatment container samples (Figure 2B). Proteobacteria, in contrast, were largely similar in their family composition once they transitioned to our experimental setup; however, treating samples with glucose (Treated container) increased the overall relative abundance of Proteobacteria, specifically through the inflation of the family Caulobacteraceae. Other families shifted slightly in composition, including slight decreases in the relative abundance of Sphingomonadaceae and an increase in Azospirillaceae and Devosiaceae. Further, proportions of low abundance Proteobacterial families (<1% relative abundance) were largely reduced in the treated condition; a treatment effect that is in line with observations of total Proteobacterial taxa abundance after glucose treatment (Figure 2C).

## 4. Discussion

The addition of glucose to Harsha Lake water in a container suppressed cyanobacteria during the entire bloom season. This data is consistent with our earlier findings [19]. However, a container experiment can never accurately replicate the dynamic conditions of a lake’s variable temperature, wave action, and light conditions. The study primarily reflects the conditions in the chamber. To try to mimic the changing conditions in the Lake, we added fresh water each week and measured some of its physical, chemical and biological parameters. The Lake water itself showed that nutrients and productivity were higher early in the summer but these led to decreases in disolved oxygen and turbidity later in the summer. In addition, the temperature and pH did not increase as expected.

In the containers themselves, two trends were observed over the course of the experiment. First, the transition of water samples from Harsha Lake into the experimental containers in the controlled environmental chamber impacted the communities present within our samples in both the presence and absence of glucose treatment. *Dolichospermum* dominated the Harsha Lake water samples collected on June 9^th^. This cyanobacterium contains gas-filled vesicles, keeping the cells near a lake’s surface. This location is where the Lake water samples were initially collected. However, once the Lake water was in the containers in the environmental chamber, the Control container became dominated by the cyanobacterium *Cyanobium. Cyanobium* tends to be more competitive than other cyanobacteria at higher temperatures and low ratios of total nitrogen (TN) to total phosphorous (TP) [40]. The environmental chamber was set at a fixed temperature of 25°C, which is higher than the water temperature of the Lake in June. Also, the ratio of TN to TP typically lowers in Harsha Lake water as the blooms season progresses [23], giving *Cyanobium* an advantage.

The second observation is that glucose addition altered the microbial composition in the Treated compared to the Control container. When filtering the samples, our observations showed that the sampled water from the glucose-treated container filtered much more slowly than the samples from the Control container. This is consistent with the denser, more opaque appearance of the treated-water samples, which could be described as a “bacterial bloom”. It appears that glucose can stimulate non-cyanobacterial growth. This effect was maintained throughout our experiment despite weekly spikes of additional fresh Lake water samples.

The most obvious result of glucose addition was a dramatic reduction in overall taxa richness, most notably by an almost complete absence of cyanobacteria. This effect, however, was accompanied by increases in proportional distributions of other phyla. Bacteriodota proliferated in early samples (June 23^rd^ and July 6^th^), while Actinobacteriota and Proteobacteria proliferated later. Within the Proteobacteria, Caulobacterales were more common in the Treated container than in the Control container. Piel *et al*. [41] found that when lake water was treated with hydrogen peroxide to control cyanobacteria, Proteobacteria increased to fill the niche created.

Caulobacterales were reported to be more common in low total N and P environments [42]. Caulobacterales and Sphingomonadales populations are promoted in higher temperatures, lower concentrations of oxygen, and increased dissolved organic matter [43]. The environmental conditions in the growth chamber, with higher temperatures, reduced wave action or oxygenation, and the addition of glucose, as dissolved organic matter, may have been responsible for their growth.

Our study had several limitations. Like any container study, the results cannot be assumed to reflect the actual physical and microbiological conditions of the Lake *in situ*. By adding freshwater samples weekly to the containers, we tried to accommodate for the changing microbial content of the Lake. The changing bacterial populations in the containers during the summer suggest this approach met with some success. However, it was not possible to simulate the physical conditions like the varying Lake water temperature, wave oxygenation, stratification, or changing light intensity, which will greatly alter conditions like disolved oxygen (DO) concentrations compare to the fixed container used, even one that is mixed daily. Although DO was not measured in this experiment, we realize this is a critical element that will need consideration in future experiments assessing practicality. The quantity of glucose added will need to be significantly reduced to prevent eutrophication and hypoxia in an aquatic ecosystem. This will likely require a slow-release glucose system targeted to the photosynthetic zone. Future experiments will test the practicality of adding glucose to control cyanobacteria using such a system.

## 5. Conclusion

The addition of glucose to a container receiving weekly additions of lake water suppressed the cyanobacterial populations during the entire summer bloom season. The glucose appears to stimulate the diversity of certain bacterial taxa at the expense of the cyanobacteria, but reduced glucose concentrations will need to be tested for practicality.

## Figures and Tables

**Table 1.** Physical, chemical and biological parameters* measured each week in the study Lake during the summer of 2022 (ND = not determined).

Date	TNH_4_ μg N/L	TNO_2–3_ μg N/L	TNO_2_ μg N/L	TRP μg P/L	BGA-PC RFU	Chlor. RFU	ODO %sat	ORP mV	Sp Cond. μS/cm	Temp. °C	Turb. FNU	pH SU

6/8	12.1	37.7	14.6	58.8	ND	ND	ND	ND	ND	ND	ND	ND
6/15	ND	ND	10.6	ND	9.9	1.0	224	80	264	29.3	14.0	9.8
6/22	9.5	7.8	12.2	40.2	6.3	1.2	244	105.1	269	29.7	9.9	9.9
6/29	ND	ND	ND	ND	1.3	0.6	117	124.8	267	27.1	4.2	9.4
7/6	8.7	44.5	ND	55.3	1.7	1.1	107	109.8	272	28.9	3.5	9.3
7/13	10.9	8.7	10.7	47.3	1.2	1.0	126	132.8	272	29.2	2.7	9.3
7/20	6.2	ND	6.6	22.9	0.8	0.7	122	101.8	274	29	1.6	9.2
7/27	10.8	16.4	ND	37.8	ND	ND	ND	ND	ND	ND	ND	ND
8/3	4.1	11.5	9.7	49.6	0.6	0.8	114	131.7	278	28.3	−0.1	9.0
8/10	ND	ND	ND	ND	0.5	1.1	106	91.9	274	28.5	1.3	8.9
8/17	4.4	8.9	8.2	50.3	0.3	0.7	91	114.7	275	27.1	0.2	8.7
8/24	3.3	19.7	8.9	72.5	−0.1	0.1	99	149.3	272	27.8	−0.6	8.7

* **TNH**_4_—total disolved amonium as μg nitrogen per liter; **TNO**_2–3_—total disolved nitrates/nitrites as μg nitrogen per liter; **TNO**_2_—total disolved nitrite as μg nitrogen per liter; **TRP**—total recoverable phosphorus; **BGA-PC**—blue-green algae as phyco-cyanin, measured in relative fluorescence units; **Chlor.**—chlorophyll-a, as measured in relative fluorescence units; **ODO**—oxygen as disolved oxygen, reading corrected with temperature and local barometric pressure at the time of calibration; **ORP**—oxidation reduction potential in millivolts (mV); **Sp. Cond.**—specific conductivity measured in micro-Siemens per centimeter (μS/cm); **Temp.**—temperature in centigrade; **Turb.**—turbidity as measured by scattered light method using Formazin Nephelometric Unit (FNU) scale; **pH**—standard unit: pH is a logarithm (the negative of the logarithm of H+ activity), and as such, it has no units.

## Data Availability

All data will be available at the NIH-PMC website. All sequence data have been deposited in the NCBI sequence read archive at accession number: PRJNA972685.
